# Point-of-care Ultrasound for Detecting Methamphetamine-associated Heart Failure in the Emergency Department

**DOI:** 10.5811/westjem.53982

**Published:** 2026-07-16

**Authors:** Madison Nashu, Jordan Lam, Zarak Khan, Soheil Saadat, Roy Almog, Ron Goubert, Joshua Wasmund, Heesun Choi, Shadi Lahham, Edmund Hsu, Megan Guy, John C. Fox

**Affiliations:** * University of California Irvine, Department of Emergency Medicine, Orange, California; † University of California Irvine School of Medicine, Irvine, California; ‡ Yale New Haven Hospital, Department of Emergency Medicine, New Haven, Connecticut; § Eisenhower Health, Department of Emergency Medicine, Rancho Mirage, California; || Kaiser Permanente, Anaheim, California

## Abstract

**Introduction:**

Methamphetamine-associated heart failure with reduced ejection fraction is a serious consequence of methamphetamine use often underrecognized in the emergency department (ED). Point-of-care ultrasound (POCUS) offers rapid, non-invasive cardiac screening for high-risk populations. This study evaluated the diagnostic yield of POCUS for detecting methamphetamine-associated heart failure with reduced ejection fraction in ED patients who use methamphetamine.

**Methods:**

We conducted this prospective cohort study between December 2020–February 2024 at an urban Level I trauma center ED. The primary outcome was diagnostic yield of cardiac POCUS for abnormal left ventricular ejection fraction (LVEF) and abnormal sex-specific left ventricular end-diastolic diameter in patients with methamphetamine use, with secondary analyses assessing associations with use duration and frequency. Diagnostic yield was calculated as the proportion of completed POCUS examinations identifying abnormalities. We used E-point septal separation to calculate LVEF; < 40% was abnormal. Left ventricular end-diastolic diameter abnormality (> 5.8 cm males, > 5.2 cm females) was categorized as mild, moderate, or severe (mild, 5.9–6.3/5.3–5.6; moderate, 6.4–6.8/5.7–6.1; severe, > 6.8/> 6.1 cm males/females). Physician-performed POCUS assessed LVEF and left ventricular end-diastolic diameter in patients with a methamphetamine use history and a comparison group of non-users.

**Results:**

Of the 136 enrolled patients, 84 (61.8%) reported methamphetamine use. Among methamphetamine users, diagnostic yield of cardiac POCUS was as follows: reduced LVEF in 22 of 70 with measurable LVEF (31.4% [20.9–43.6%]); any sex-specific left ventricular end-diastolic diameter abnormality in 30 of 84 (35.7% [25.6–46.9%]); and severe sex-specific left ventricular end-diastolic diameter abnormality in 15 of 84 (17.9% [10.4–27.7%]). Corresponding values in non-users were 3 of 52 (5.8% [1.2–16.0%]; *P* < .001), 10 of 52 (19.2% [9.6–32.5%]; *P* = .05), and 3 of 52 (5.8% [1.2 – 16.0%]; *P* = .07), respectively. Longer duration (odds ratio [OR] 6.05, 95% confidence interval [CI] 1.46–25.10) and higher frequency (OR 5.93, 95% CI, 1.52–23.06) of methamphetamine use were associated with reduced LVEF.

**Conclusion:**

Cardiac point-of-care ultrasound demonstrated a clinically significant diagnostic yield in detecting methamphetamine-associated heart failure with reduced ejection fraction among methamphetamine users in the ED, suggesting POCUS could aid early detection in this population, potentially streamlining management before disease progression.

## INTRODUCTION

Methamphetamine is an addictive central nervous system stimulant that is widely abused in the United States, with the National Survey on Drug Use and Health (NSDUH) reporting over 2.7 million people ≥ 12 years of age with usage in 2022.^1^ The abuse of this highly addictive substance has become increasingly prevalent, with an increase of 231% in hospital admissions from 2008 to 2020.^2^ Approximately 1.8 million people who reported methamphetamine use on the NSDUH met diagnostic criteria for methamphetamine use disorder.^1^ Methamphetamine use disorder is a rapidly developing condition marked by cycles of heavy methamphetamine use followed by periods of abstinence. It results in serious medical consequences that are difficult to treat, such as cognitive, neurological, cardiovascular, and cerebrovascular conditions, and can lead to death.^3^ An increased risk is seen in males, those without a high school degree, individuals who earn < $20,000 annually, and those with Medicaid only or no insurance.^4^,^5^ Given the high rate of methamphetamine-associated morbidity, increasing usage rates are highly likely to increase methamphetamine-related morbidity, emergency department (ED) visits, and healthcare costs.

One of the most dangerous consequences of methamphetamine use is systolic heart failure, traditionally characterized by reduced left ventricular ejection fraction (LVEF), increased left ventricular mass index, and increased end-diastolic volume index.^6^–^8^ Heart failure of this etiology is known as methamphetamine-associated heart failure with reduced ejection fraction. As with other forms of heart failure, optimal management and reduction in morbidity and mortality require early diagnosis, accurate assessment of left ventricular function, and timely intervention. 9

The ED often serves as the entryway to the healthcare system for methamphetamine users, typically during acute methamphetamine-related medical emergencies. As many of these patients are frequently discharged without a hospital admission, the ED may be their only interaction with the healthcare system.^10^ However, because methamphetamine users often present to the ED with non-cardiac complaints, including altered level of consciousness, skin infections, abdominal pain, suicide attempts, psychosis and/or trauma,^11^ underlying cardiac dysfunction may be underrecognized in this setting.

The risk of missed identification of this type of systolic heart failure underscores the need for efficient, accessible tools to screen for cardiac dysfunction in methamphetamine users. Compared with formal echocardiography, point-of-care ultrasound (POCUS) provides a rapid and more readily accessible assessment of cardiac function at bedside in the ED and may serve as a valuable tool for screening early-onset heart failure in methamphetamine users. Despite its potential utility, the diagnostic yield of POCUS in identifying methamphetamine-associated heart failure with reduced ejection fraction in this high-risk population has not been previously studied. Therefore, in this study we aimed to determine the diagnostic yield of POCUS in detecting abnormal LVEF and abnormal left ventricular end-diastolic diameter by sex in methamphetamine users presenting to the ED, compared to non-users.

**Population Health Research Capsule**

What do we already know about this issue?

*Methamphetamine use is associated with systolic heart failure with reduced left ventricular ejection fraction (LVEF)*.

What was the research question?

*What is the diagnostic yield of point-of-care ultrasound (POCUS) for detecting heart failure in emergency department patients who use methamphetamine?*

What was the major finding of the study?

*The diagnostic yield of cardiac POCUS in methamphetamine users was 31.4% (20.9–43.6%) for reduced LVEF*.

How does this improve population health?

*Cardiac POCUS may serve a public health role by enabling early heart failure detection in methamphetamine users*.

## METHODS

### Study Design and Participant Selection

We conducted this prospective cohort study at an urban, Level I trauma center within an academic quaternary-care facility that hosts an established emergency medicine residency and emergency ultrasound fellowship training program. Trained research associates (RA) screened and enrolled a convenience sample of adult participants who presented to the ED between December 2020–February 2024. Patients who reported a history of methamphetamine use during their ED visit were eligible for inclusion in the methamphetamine-user group, regardless of their chief complaint. The non-user group was recruited from the general ED population to allow assessment of POCUS findings in patients who denied methamphetamine use or had no prior methamphetamine-positive urine toxicology screen. The RAs enrolled a convenience sample of these participants, regardless of their presenting complaint, provided their presentation did not meet any exclusion criteria. Exclusion criteria included individuals who were pregnant or found to be pregnant, as well as those with a history of heart failure unrelated to methamphetamine use, peripartum cardiomyopathy, coronary artery disease, valvular diseases, previous heart transplant, left ventricular assist devices, congenital heart failure, moderate to severe trauma, acute respiratory failure, or known malignancy. Only participants with the capacity to understand and sign written informed consent in English were eligible for inclusion in the study.

The POCUS examinations were performed by ultrasound-trained attending physicians and resident physicians in the ED using the Mindray TE7 ultrasound system or TE X portable ultrasound system (Mindray DS USA Inc., Mahwah, NJ) with a phased-array 2–5 megahertz transducer on preprogrammed cardiac settings. The study protocol was reviewed and approved by the University of California, Irvine Institutional Review Board.

### Data Collection

After obtaining informed consent, trained RAs collected demographic and clinical information from patients through a survey. This included age, sex, history of controlled and uncontrolled hypertension, history of prior and current methamphetamine use, history of tobacco smoking, and history of other recreational drug use. Patients who reported positive tobacco, methamphetamine, or other drug use were further queried about the frequency and duration of their use.

Following the survey, POCUS examinations were performed during the patient’s ED stay. Some patients were acutely intoxicated; others had a history of methamphetamine use without current intoxication, and some had no history of methamphetamine use. Patients were positioned in the supine or left lateral decubitus position for the cardiac POCUS to be performed. Trained emergency physicians obtained a parasternal long-axis view. In this view, the physician measured the E-point septal separation using M-mode. This approach was chosen because it enables convenient assessment of left ventricular systolic function in time-sensitive settings and has been found to be associated with LVEF, providing a rapid, reliable assessment for left ventricular systolic function, particularly in emergency settings.^12^ The E-point is the maximum anterior motion of the anterior mitral valve leaflet during early diastole. It is seen as the highest peak on the M-mode tracing of the mitral valve. The physician obtained this tracing by placing the cursor over the distal tip of the anterior leaflet of the mitral valve. To determine the E-point septal separation, the ultrasound calipers were used to measure the distance between the E-point and the interventricular septum on the tracing.

We calculated the LVEF using the following formula: LVEF = 75.5 - [2.5 * (*E-point septal separation in mm*)]. Subsequently, maintaining the parasternal long-axis view, physicians measured the left ventricular end-diastolic diameter using M-mode by placing the cursor just outside the anterior leaflet of the mitral valve to obtain another tracing. The ultrasound calipers were used to measure the distance between the interventricular septum and the inner surface of the wall of the left ventricle. The LVEF values < 40% were considered abnormal. We defined left ventricular end-diastolic diameter abnormality by sex as end diastolic diameter of left ventricle > 5.8 cm in males and 5.2 cm in females. Left ventricular end-diastolic diameter values were further categorized as mild (5.9–6.3 cm for males; 5.3–5.6 cm for females), moderate (6.4–6.8 cm for males; 5.7–6.1 cm for females), and severe (> 6.8 cm for males; > 6.1 cm for females). These cutoff values are defined by the American Society of Echocardiography.^13^

### Statistical Analysis

We estimated the sample size based on power (0.9) to detect an *R*^2^ of 0.1 attributed to two independent variable(s) using an *F*-test with a significance level (alpha) of 0.05. The variables tested are adjusted for an additional two covariates that have a combined *R*^2^ of 0.1 by themselves. The effect size was determined conservatively. We used Power Analysis and Sample Size Software v19.01 (*NCSS, LLC*. Kaysville, UT) for sample size calculations. The diagnostic yield was calculated as the proportion of completed POCUS examinations that identified the abnormality of interest.^14^

Frequencies were presented as counts and percentages. We used comparisons across categories of nominal variables using the Pearson chi-square test or Fisher exact test. Comparisons across ordinal variables were conducted using the Mantel chi-square test for trend. For multivariable analysis, we used logistic regression to find variables associated with reduced LVEF (coded as normal or reduced) and ordinal regression to assess left ventricular end-diastolic diameter abnormality for sex (categorized as normal, mild/moderate abnormality, severe abnormality). Analyses were performed using IBM SPSS Statistics for Windows v28.0 (International Business Machines Corporation, Armonk, NY). A *P* value < .05 was considered statistically significant.

## RESULTS

### Study Sample

We enrolled 84 patients with self-reported methamphetamine use in the user group, and 52 patients with no history of methamphetamine use in the non-user group. Sixty-two (73.8%) patients in the user group and 38 (73.1%) in the non-user group were male (Table 1). Of the 84 participants with self-reported methamphetamine use, LVEF measurements were available for 70, with 14 examinations excluded due to incomplete or technically inadequate imaging. The LVEF measurements were available for all 52 participants in the non-user group.

The distribution of smoking history (*n* = 75, 55.1%) and hypertension (*n* = 46, 33.8%) was similar between men and women (*P* = .44 and .29, respectively). The distribution of reduced LVEF (*n* = 25, 20.5%) and abnormal left ventricular end-diastolic diameter by sex (*n* = 40, 29.4%) did not differ significantly between sexes (*P* = .19 and .20, respectively) (Table 1).

### Point-of-care Ultrasound Characteristics

Among 122 subjects with LVEF data, mean LVEF was 54.0% (17.0) (median 60%, interquartile range 48–65.5). Mean LVEF was 54.9% (17.0) in men and 51.2% (16.8) in women. Among 136 participants with left ventricular end-diastolic diameter data, mean left ventricular end-diastolic diameter was 4.9 (1.8 cm), 5.1 (1.8) cm in men vs 4.4 (1.7) cm in women.

### Diagnostic Yield

The diagnostic yield of cardiac POCUS for detecting abnormalities in the user group was as follows: reduced LVEF in 22 of 70 subjects (31.4%; 20.9–43.6%); any sex-specific left ventricular end-diastolic diameter abnormality in 30 of 84 (35.7%; 25.6–46.9%); and severe sex-specific left ventricular end-diastolic diameter abnormality in 15 of 84 (17.9%; 10.4–27.7%). Corresponding values in non-users were 3 of 52 (5.8%; 1.2–16.0%; *P* < .001), 10 of 52 (19.2%; 9.6–32.5%; *P* = .05), and 3 of 52 (5.8%; 1.2–16.0%; *P* = .07), respectively.

### Association Between Methamphetamine Use and Reduced Left Ventricular Ejection Fraction

In a univariable analysis, methamphetamine use was associated with reduced LVEF (Table 2, *P* < .001). Reduced LVEF was identified in 22 of 70 participants in the user group (31.4%) compared with 3 of 52 participants in the non-user group (5.8%) (Table 2; *P* < .001). The LVEF could not be confirmed for 14 participants in the user group. Reduced LVEF was more common with increasing duration and frequency of methamphetamine use (both *P* < .001) (Table 2).

In logistic regression analysis, reduced LVEF was associated with methamphetamine use *(P* = .02, odds ratio [OR] 5.03) and hypertension (*P* = .02, OR 3.32). Age, sex, and smoking failed to retain statistical significance and were excluded from the final model (Table 3). Longer duration (OR 6.05, 95% CI, 1.46–25.10) and higher frequency (OR 5.93, 95% CI, 1.52–23.06) of methamphetamine use were associated with reduced LVEF after adjusting for hypertension.

### Association Between Left Ventricular End-diastolic Diameter Abnormality by Sex and Methamphetamine Use

In univariable analysis, methamphetamine use was associated with an abnormal left ventricular end-diastolic diameter by sex (*P* = .02) (Table 2). Abnormal left ventricular end-diastolic diameter was observed in 30 of 84 participants (35.7%) in the user group and 10 of 52 participants (19.3%) in the non-user group. This difference was statistically significant (*P* = 0.02) (Table 2). The duration of methamphetamine use was also associated with an abnormal left ventricular end-diastolic diameter by sex, indicating that left ventricular end-diastolic diameter abnormalities increased with prolonged methamphetamine use (*P* < .001) (Table 2). A statistically significant association between the self-reported frequency of methamphetamine use per week and abnormal left ventricular end-diastolic diameter by sex was not observed (*P* = .08) (Table 2).

We assessed the association between abnormal left ventricular end-diastolic diameter by sex and the duration of methamphetamine use using ordinal regression analysis. Years of methamphetamine use and hypertension (P = .01) were found to be associated with abnormal left ventricular end-diastolic diameter by sex (Table 4).

## DISCUSSION

We observed a consistent association between methamphetamine use and early-onset heart failure. The diagnostic yield of POCUS for detecting indicators of methamphetamine-associated heart failure with reduced ejection fraction—31.4% for LVEF abnormality, 35.7% for sex-specific left ventricular end-diastolic diameter abnormality, and 17.9% for severe left ventricular end-diastolic diameter abnormality—represents a clinically significant rate of detection in a population that is not routinely screened for cardiac dysfunction. These findings underscore the potential value of using cardiac POCUS to screen for heart failure of this etiology in methamphetamine users who present to the ED, regardless of their chief complaint.

The high diagnostic yield of POCUS highlights the need for increased awareness among emergency physicians regarding the cardiovascular risks of methamphetamine use, especially in geographical areas where methamphetamine use is prevalent. Notably, patients at risk for methamphetamine-associated heart failure with reduced ejection fraction may not present with typical heart failure symptoms.

Methamphetamine users often present with non-cardiac complaints, such as altered mental status, skin infections, abdominal pain, suicide attempts, or trauma,^11^ yet remain at a significantly elevated risk for heart failure compared to non-users. Many of the patients in our sample interacted with the healthcare system solely through the ED and were frequent utilizers, suggesting they may lack access to consistent preventive care. Thus, greater awareness of the risks associated with methamphetamine-associated heart failure with reduced ejection fraction should be accompanied by targeted ED screening protocols for this etiology of systolic heart failure.

Screening methamphetamine-users in the ED with cardiac POCUS may offer an accessible strategy for the early identification of methamphetamine-associated heart failure with reduced ejection fraction, even in the absence of overt heart failure symptoms. Identification of cardiac dysfunction through POCUS screening may prompt further evaluation, including comprehensive echocardiography, B-type natriuretic peptide testing, and troponin measurements, as well as the initiation of medical management, such as antihypertensive or diuretic therapy. It also creates additional opportunities for engaging patients in social work and substance use disorder treatment services. Given the increasing availability of POCUS in emergency departments and the high proficiency of emergency physicians in its use,^15^,^16^ integrating POCUS into ED screening protocols for methamphetamine users represents a feasible and potentially high-impact strategy for mitigating the burden of methamphetamine-associated heart failure with reduced ejection fraction.

This study also observed a dose-response relationship between the frequency and duration of methamphetamine use and heart failure severity. This relationship remains statistically significant after adjusting for hypertension. The observed dose-response relationship is consistent with one of Hill’s criteria for causality (17), supporting an association between greater methamphetamine exposure and more severe cardiac dysfunction. Furthermore, the biological plausibility of these findings is well-supported by existing literature,^18^–^20^ which outlines the well-known cardiovascular toxic effects of methamphetamine, reinforcing the idea that increased use has a dose-dependent effect on the severity of heart failure.

Although the exact mechanisms behind the development of methamphetamine-associated heart failure with reduced ejection fraction remain unclear, several factors are believed to contribute to its development. These include increased free radical production leading to oxidative stress, elevated p53 activity that promotes apoptosis, mitochondrial dysfunction, altered gene expression, and disruptions in intracellular calcium homeostasis.^20^ These pathological processes impair contractile function by disrupting electrical-mechanical coupling, calcium signaling, and increasing inflammation. Over time, chronic inflammation, myocyte loss, and fibrosis lead to dilated cardiac chambers and reduced systolic function, resulting in dilated cardiomyopathy and heart failure with reduced ejection fraction.^18^,^19^ Additionally, methamphetamine-induced sympathetic activation causes prolonged periods of hypertension and tachycardia, which can further exacerbate methamphetamine-associated heart failure with reduced ejection fraction.^19^ Notably, it has been shown that the condition is reversible if methamphetamine use ceases before cardiac fibrosis develops.^18^,^21^ This, combined with our finding of a dose-response relationship between methamphetamine use and increased severity of heart failure, underscores the importance of early detection and intervention, particularly in the ED, where methamphetamine users frequently present.^22^

The observed dose-response relationship between frequency and duration of methamphetamine use and heart failure severity may provide an opportunity to counsel patients that increased dose and frequency of use can lead to worsening heart failure, even if they are not yet experiencing typical heart failure symptoms. Additionally, these findings suggest that, among individuals with methamphetamine use disorder, reduction in dose and frequency, even without complete cessation, may be associated with improved cardiovascular outcomes and slower disease progression. Such education, combined with interventions such as substance use counseling, community resources, exercise, and closer follow-up, can help to reduce long-term cardiovascular risk and encourage positive behavioral changes. Additionally, providing access to psychosocial interventions such as contingency management, which promotes reduction in substance use and abstinence through positive reinforcement, and cognitive behavioral therapy, which targets harmful thought patterns, can be effective in reducing methamphetamine use and supporting long-term recovery.^23^

While this study has multiple implications for further work in emergency medicine and cardiology, an alternative potential direction for future research could be to explore the use of handheld or cart-based ultrasound devices in primary care settings or substance use disorder treatment centers. Given that the Accreditation Council for Graduate Medical Education has made POCUS a requirement in family medicine residency programs,^24^ there is greater potential for POCUS in outpatient settings to monitor improvements in LVEF, left ventricular end-diastolic diameter, and cardiac dilation over time. This approach offers a promising opportunity for early detection, intervention, and proactive cardiovascular disease monitoring among methamphetamine users in outpatient care settings. Future work should also evaluate the cost-effectiveness, feasibility, and optimal implementation strategies of POCUS screening in these non-ED settings to ensure broader applicability and sustainability. This work also encourages collaboration between the specialties of cardiology and emergency medicine, perhaps indicating a need for easy access to outpatient clinic services for those in our community most at risk for undiagnosed and untreated methamphetamine-associated heart failure with reduced ejection fraction. Ultimately, integrating cardiac POCUS into both acute and preventive care pathways may not only improve the early detection of this condition but also serve as a critical touch point for engaging patients in treatment and mitigating the long-term consequences of methamphetamine use.

## LIMITATIONS

In this prospective study, we enrolled a convenience sample of patients from a single large academic medical center in Orange County, California. Patients were enrolled from 8 am to midnight, meaning that those who presented outside this time range were not enrolled. Given that the consent information was available in English, non-English-speaking patients were not included in the study population. These factors may have introduced selection bias, affecting both methamphetamine-user and non-user groups, which potentially limit generalizability. The POCUS examinations were performed by multiple trained physicians; however, interobserver variability was not formally assessed. Physicians performing POCUS were not blinded to patient exposure status, which could have introduced measurement bias. Formal echocardiography was not systematically performed; thus, POCUS findings were not validated against a gold standard. Additionally, this study was not designed to distinguish acute methamphetamine-induced cardiac effects from chronic cardiomyopathy.

While we were able to control for confounders such as hypertension and smoking, we were unable to control for other potential confounders such as additional substance use, which may influence LVEF. The accuracy of the data associated with dose dependency is related directly to our patients’ responses. Recall bias may affect these responses, especially for events in the distant past. Additionally, many enrolled patients suffer from polysubstance use disorder, which may have impaired their recall of methamphetamine use duration and frequency. Self-reporting bias is also likely when evaluating reported data associated with a highly stigmatized substance. Lastly, we had a limited sample size of patients with severely reduced ejection fraction, likely due to the high mortality rates associated with the severe cardiomyopathy seen in patients with higher frequency and duration of methamphetamine use.

## CONCLUSION

Methamphetamine use was consistently associated with early-onset heart failure, and cardiac POCUS demonstrated a clinically meaningful diagnostic yield in this population. While these findings suggest POCUS could aid in early detection among methamphetamine users presenting to the ED, further multicenter studies are needed to assess cost effectiveness, feasibility, and long-term outcomes before broader implementation.

## Figures and Tables

**Table 1:** Demographic and point-of-care ultrasound (POCUS) characteristics in a prospective study of POCUS screening for methamphetamine-associated heart failure.

	Sex				Total	

	Male		Female			
		
	Count	Percentage	Count	Percentage	Count	Percentage
Age group						
≤ 35	50	50.0%	11	30.6%	61	44.9%
36 – 45	14	14.0%	13	36.1%	27	19.9%
46 – 55	22	22.0%	7	19.4%	29	21.3%
≥ 56	14	14.0%	5	13.9%	19	14.0%
Smoking history						
No	44	44.0%	17	47.2%	61	44.9%
Yes	56	56.0%	19	52.8%	75	55.1%
Hypertension						
No	68	68.0%	22	61.1%	90	66.2%
Yes	32	32.0%	14	38.9%	46	33.8%
Methamphetamine						
No	38	38.0%	14	38.9%	52	38.2%
Yes	62	62.0%	22	61.1%	84	61.8%
Methamphetamine use:						
Years of use						
0	38	38.8%	14	38.9%	52	38.8%
1 – 10	36	36.7%	8	22.2%	44	32.8%
≥ 11	24	24.5%	14	38.9%	38	28.4%
Frequency per week						
0	38	39.2%	14	38.9%	52	39.1%
1 – 3	23	23.7%	6	16.7%	29	21.8%
≥ 4	36	37.1%	16	44.4%	52	39.1%
LVEF abnormality						
Normal	76	82.6%	21	70.0%	97	79.5%
Abnormal	16	17.4%	9	30.0%	25	20.5%
LVEDD abnormality by sex						
Normal	70	70.0%	26	72.2%	96	70.6%
Mild/Moderate	19	19.0%	3	8.3%	22	16.2%
Severe	11	11.0%	7	19.4%	18	13.2%

Any differences in denominators between rows are due to missing values for the respective variable, including 14 inadequate LVEF images in the methamphetamine-user group.

Percentages represent proportions within each sex category.

*LVEF*, left ventricular ejection fraction; *LVEDD*, left ventricular end-diastolic diameter.

**Table 2:** Associations of point-of-care ultrasound-measured left ventricular ejection fraction abnormality and left ventricular end-diastolic diameter abnormality by sex with methamphetamine use, duration, and frequency.

		Methamphetamine use					Years of methamphetamine use							Frequency of methamphetamine use per week						
		
		Non-user		User		*P* value	0		1 – 10		≥ 11		*P* value	0		1 – 3		≥ 4		*P* value

		*N*	(%)	N	(%)		*N*	(%)	N	(%)	N	(%)		N	(%)	N	(%)	N	(%)	
LVEDD abnormality by sex	Normal	42	80.8	54	64.3	.024*	42	80.8	31	70.5	21	55.3	.002*	42	80.8	17	58.6	34	65.4	.077*
	Mild/Moderate	7	13.5	15	17.9		7	13.5	9	20.5	6	15.8		7	13.5	5	17.2	10	19.2	
	Severe	3	5.8	15	17.9		3	5.8	4	9.1	11	28.9		3	5.8	7	24.1	8	15.4	
LVEF abnormality	Normal	49	94.2	48	68.6	<.001**	49	94.2	26	72.2	21	65.6	<.001*	49	94.2	16	76.2	31	66.0	<.001*
	Abnormal	3	5.8	22	31.4		3	5.8	10	27.8	11	34.4		3	5.8	5	23.8	16	34.0	

* Based on Mantel-Haenszel chi-square test for trend.

** Based on Fisher exact test.

Denominators for percentages can be calculated by summing the *n* values within each category. Denominators differ between LVEDD and LVEF due to 14 inadequate images in the methamphetamine-user group for LVEF.

*LVEF*, left ventricular ejection fraction; *LVEDD*, left ventricular end-diastolic diameter; *POCUS*, point-of-care ultrasound.

**Table 3:** Association of methamphetamine use, frequency, and duration with reduced left ventricular ejection fraction, adjusted for hypertension—logistic regression analysis.

Models	Variables in the model	B	*P*	OR	95%	CI
Model 1: Methamphetamine use	Hypertension	1.199	.017	3.315	1.235	8.897
	Methamphetamine use	1.615	.017	5.028	1.341	18.853
	Model constant	−3.044	<.001	N/A	N-A	N/A
Model 2: Years of methamphetamine use	Hypertension	1.126	.028	3.085	1.131	8.412
	Years of methamphetamine use (reference: 0)					
	Years of methamphetamine use: 1–10	1.434	.050	4.194	0.997	17.633
	Years of methamphetamine use: >10	1.800	.013	6.049	1.458	25.098
	Model constant	−3.024	<.001	N/A	N-A	N/A
Model 3: Frequency of methamphetamine per week	Hypertension	1.147	.026	3.149	1.149	8.627
	Frequency of methamphetamine use per week (reference: 0)					
	Frequency of methamphetamine use per week: 1–3	1.179	.152	3.251	0.649	16.287
	Frequency of methamphetamine use per week: >3	1.779	.010	5.925	1.522	23.060
	Model constant	−3.029	<.001	N/A	N-A	N/A

*LVEF*, left ventricle ejection fraction; *OR*, odds ratio.
